# Cortisol reactivity in patients with anorexia nervosa after stress induction

**DOI:** 10.1038/s41398-020-00955-7

**Published:** 2020-08-10

**Authors:** Ileana Schmalbach, Benedict Herhaus, Sebastian Pässler, Sarah Runst, Hendrik Berth, Silvia Wolff-Stephan, Katja Petrowski

**Affiliations:** 1 Medical Psychology and Medical Sociology, University Medical Center of the Johannes Gutenberg-University, Mainz, Germany; 2 Technische Universität Dresden, Carl Gustav Carus Faculty of Medicine, Division of Psychological and Social Medicine and Developmental Neurosciences, Research Group Applied Medical Psychology and Medical Sociology, Dresden, Germany; 3 University Hospital Carl Gustav Carus Dresden, Department of Psychotherapy and Psychosomatic Medicine, Dresden, Germany

**Keywords:** Predictive markers, Human behaviour

## Abstract

There is a need of experimental studies on biomarkers in patients with anorexia nervosa (P_AN_), especially in the context of stress, in order to foster understanding in illness maintenance. To this end, the cortisol response to an acute stressor was investigated in *n* = 26 P_AN_ (BMI: 19.3 ± 3.4 kg/m^2^), age, and gender matched to *n* = 26 healthy controls (HC; BMI: 23.08 ± 3.3 kg/m^2^). For this purpose, salivary cortisol parameters were assessed in two experimental conditions: (1) rest/no intervention and (2) stress intervention (TSST; Trier Social Stress Test). In addition, psychological indicators of stress were assessed (Primary Appraisal Secondary Appraisal, Visual Analogue Scale, and Trier Inventory for the assessment of Chronic Stress), as well as psychological distress, depression, and eating disorder (ED) symptoms. A 2 × 2 × 8 ANOVA demonstrated elevated cortisol levels in P_AN_ in the resting condition. In the stress intervention no significant *group* effect in terms of *cortisol* (*F* (1, 50) = 0.69; *p* = 0.410; $\eta _p^2 = 0.014$). A significant *condition* (*F* (1, 50) = 20.50; *p* = 0.000; $\eta _p^2 = 0.291$) and *time* effect (*F*(2.71, 135.44) = 11.27; *p* = 0.000; $\eta _p^2 = 0.20$) were revealed, as well as two significant interaction effects. First: *Condition* × *group* (*F* (1, 50) = 4.17, *p* = 0.046; $\eta _p^2 = 0.077$) and second: *Condition* × *time* (*F* (2.71, 135.44) = 16.07, *p* = 0.000, $\eta _p^2 = 0.24.$). In terms of AUC_G_, no significant differences between both groups were exhibited. Regardless, significant results were evinced in terms of an increase (AUC_i_: *F*(1, 50) = 20.66, *p* = 0.015, $\eta _p^2 = 0.113$), baseline to peak (+20 min post-TSST: *t*_5_ = 16.51 (9.02), *p* = 0.029) and reactivity (*M*_PAN_ = 0.73 vs. *M*_HC_ = 4.25, *p* = 0.036). In addition, a significant correlation between AUC_G_ and BMI: *r* (24) = −0.42, *p* = 0.027 was demonstrated, but not between AUC_i_ and BMI (*r* (24) = −0.26, *p* = 0.20). Psychological indices suggested higher levels of chronic and perceived stress in P_AN_ relative to HC. However, stress perception in the stress condition (VAS) was comparable. Additional analyses demonstrated that ED-symptoms are highly correlated with psychological distress and depression, but not with BMI. In addition, it could be demonstrated that reactivity is rather related to ED-symptoms and psychological burden than to BMI. In conclusion, P_AN_ showed elevated basal cortisol levels at rest and exhibited a blunted cortisol reactivity to the TSST as evinced by salivary cortisol parameters. Further, it was shown that weight recovery influences reversibility of hypercortisolemia, i.e., cortisol levels normalize with weight gain. However, HPAA (hypothalamus–pituitary–adrenal axis) irregularities in terms of reactivity persist even at a BMI ≤ 19.3 (±3.4). Our data suggest that pronounced psychological burden in P_AN_, have a greater impact on the HPAA functionality (secondary to the ED) than BMI itself.

## Introduction

A growing plethora of studies has increasingly classified eating disorders (EDs) as a relevant cause of mortality, particularly in the case of anorexia nervosa (AN), since it has the highest mortality rate among EDs^1^. Standardized mortality ratios show at least five times greater rate of death in AN than in the general population^1^. Only less than half of P_AN_ recovered at follow-up due to severe effects of illness progression on both, mental and physical health^2^. Maintenance of underweight, fear of weight gain, purging behaviors, constant weight and shape concerns as well as emotional dysregulation are typical characteristic of P_AN_^3–5^. Even though, the etiopathogenesis of EDs is still unclear, the role of the HPAA axis^6^ and stressful life events have been emphasized in the onset and maintenance of EDs^7–13^. The sympathetic nervous system and the hypothalamus–pituitary–adrenal axis (HPAA) work as mediators of the stress response. Stress activates the HPAA altering cortisol levels as a regulatory function (e.g.,^14,15^). Cortisol levels gradually increase within a few minutes (~10 min) after stimulation and peaks between 10–30 min. after stress termination^16^ for details on normal function see^14,17^. The detection of adequate cortisol release results in a negative feedback loop signaling cessation of cortisol release. This termination is crucial for limiting cortisol exposure in the organism, since chronic exposure is detrimental to health^18^ and may contribute to the pathophysiology of anxiety and mood disorders^19–21^. The system can drift from normal, adaptive HPAA functioning in a variety of ways, e.g., by not activating the system when necessary, activating it when dispensable, or not halting it when a stressor is adequately addressed^22^. An altered HPAA functionality has been mainly observed in individuals, who were exposed to adversity, specially at early life stages^14,23^. Alterations in P_AN_ manifest in hypercortisolemia (e.g.,^10,11^) disturbed physiological and behavioral processes^24^ related to appetite^25^ and energy expenditure^26^ and a diminished sensitivity of the hypothalamic and pituitary centers to negative feedback, as evinced in reduced suppression of cortisol after a dexamethasone-suppression test (DST^27–29^) and a blunted response to acute stress exposure^28,30–34^. HPAA dysregulation in P_AN_ has been also associated with childhood adversities^23,35^ and psychological variables, such as pronounced ED-symptomatology, body-image concerns, and psychopathology (e.g., depression, distress^36–41^). It is thought that these variables induce constant alertness and stress influencing HPAA functioning^35,42^. In general, cortisol plays an essential role in promoting allostasis, including mediating and suppressing healthy stress responses^15^. On the other hand, chronic exposure to stress has detrimental effects on health and may contribute to the pathophysiology of affective disorders^19–21^.

In research related to P_AN_ dominates a great consensus on HPAA hyperactivity (e.g., hypercortisolemia^10,28,43–46^) at rest, mainly accounted by the well-known effects of starvation and weight loss on HPAA function^6,31^. Södersten et al.^47^ even claim that weight related issues, e.g., eating behavior and extreme caloric restriction are major causes for the physiological and psychological changes in P_AN_. This phenomenon has been additionally supported by research on healthy individuals^31,48^. An earlier experimental study reported that even healthy individuals, who starve develop similar psychological and physiological symptoms characteristic to AN. Also, in recent years, numerous studies on volunteers free of an ED evinced that extreme caloric restriction (e.g., fasting) resulted in elevated cortisol compared with less drastic diets^48–51^. Cortisol levels and length of caloric restriction were negative correlated, suggesting an initial increase in cortisol, but a decrease (to baseline) after several hours (e.g.,^48,52^). This outcome implies, that excessive caloric deprivation results in a temporary HPAA hyperactivity in healthy individuals^48^. These studies underline the importance of energy deprivation in the development of anorectic symptoms^53^. In conclusion, elevated baseline cortisol levels are likely observed during extreme caloric restriction in both, HC and P_AN_. However, hypercortisolemia in HC is temporary. Conversely, reversibility of HPAA dysregulation even in recovered P_AN_ is not always observed. In this regard, some studies reveal that irregularities remain even after weight restorage^54–56^. In treated/weight recovered P_AN_, baseline abnormalities in cortisol levels^28,54,56^, and menstrual cycle^57^ persisted compared with controls. To counterbalance, literature suggesting otherwise is dominant. Researchers have recurrently demonstrated that HPAA functionality is normalized with weight recovery or treatment. A great body of past and current studies^29,58^ have namely reported comparable cortisol levels (plasma, salivary) between treated/weight restored patients and controls^45,46,59–62^ and normalization of other hormones (e.g., leptin, insulin, cortisol^63^); emphasizing restoration of normal HPAA activity^64^. Due to the dominance of a variety of studies evincing normalization of HPAA dysregulation, there is a high probability that most of the endocrine functions are restored after therapy/weight recovery. Briefly, predominant literature suggests higher basal cortisol levels (at rest) in P_AN_ compared with HC. Abnormal HPAA activity such as hypercortisolemia is a.o., related to starvation. A great body of literature supports that HPAA irregularities secondary to starvation are temporary in healthy individuals and reversible in P_AN_ after treatment/weight gain. Nevertheless, HPAA activity in terms of the cortisol response to a particular stressor (i.e., reactivity) research often suggests a blunted cortisol response in P_AN_^28,32–34^. It is suggested that chronic stress exposure is linked to HPAA hyporeactivity^65,66^. Even though laboratory induced stress to investigated the HPAA activity has received attention, there are very few experimental studies on the stress response in ED patients using salivary cortisol assessments in the context of psychosocial stress (e.g., TSST). Thus, when looking at the HPAA reactivity outcomes are less abundant and less consistent. In addition, these few studies reveal contradictory results^32,67–69^. What is more, comparable experimental research on psychosocial stress has focused on bulimia nervosa, binge eating disorder (e.g.,^67,70^), or mixed groups^32,68,71^, while outcomes specific to P_AN_ have received little attention. In addition, disorder-specific samples of anorectic adults are still limited in terms of availability as well as in size (e.g.,^32,68^) and there is no clear consensus emerging from these studies. The current state of experimental research on P_AN_ in terms of the cortisol reactivity to acute psychosocial stress, even by the employment of the same protocol (TSST; Trier Social Stress Test^72^), and same hormonal parameter (i.e., salivary cortisol) is still inconclusive. For example, comparable studies on P_AN_ report a blunted stress response to the TSST^32,34,67^ relative to the control group. On the other hand, other studies could not replicate these results, e.g., the study of Monteleone et al.^68^ described a preserved HPAA activity in P_AN_ in response to the TSST. In a systematic review^73^ no differences between patient groups (incl. AN) and controls were further observed. Vocks et al.^33^ also report similarities between ED patients and HC in terms of reactivity, after a mirror exposure task. Given the inconsistencies concerning cortisol reactivity, additional research is needed in order to extend these results and foster understanding on underlying mechanisms. Differences in sample size and population, BMI, treatment status, menstrual cycle, as well as comorbidities, may explain some variation. In terms of sample size, Monteleone et al.^68^ included only seven patients with AN (vs. eight HC) making findings susceptible to random effects, due to the small sample size. In terms on weight/nutrition and therapy status, some participants were inpatients^32^, while others outpatients with unknown ED-symptoms, treatment and nutrition status^68^. In addition, Het et al.^32^ examined P_AN_ separately, while the results of Monteleone et al.^71^ refer to patients with an ED as one entity. Furthermore, some patients exhibited greater symptom severity and had comorbid depression and anxiety^32^, whereas in the study of Monteleone et al.^68^ these variables were not reported, limiting the comparability of results. The study of Het et al.^32^ appears to have the highest experimental standards, although total cortisol output in terms of AUC_G_ is not explicitly reported. Still, in this research all confounders were controlled, the stress intervention and cortisol assessment were distinctively standardized. What is more, multiple parameters besides salivary cortisol were considered (i.e., heart rate variability, salivary alpha-amylase) increasing the validity of results. In addition, it provided the largest P_AN_-sample and information on treatment, nutrition and symptom severity, which allows comprehensive conclusions.

### Purpose of the study and hypotheses

In summary, experimental studies on the cortisol reactivity specific to P_AN_ are limited and outcomes are ambiguous. Differences in sample size and population compromises generalizability of the results even among equivalent studies using the same protocol (TSST) for stress-induction. Therefore, the main objective of this study is to extend the experimental outcomes in the field of EDs in terms of the cortisol response in P_AN_ after acute stress exposure. For this purpose, we aim to provide a research with a gender and age-matched sample of adults under highly standardized conditions with the following aims. First, investigate the cortisol stress response pattern in a bigger sample (P_AN_ vs. HC) in an experimental setting. Second, examine the relationship between BMI and cortisol response. For this purpose, participants were classified according to ICD-10 in normal-weight (BMI ≤ 25 kg/m^2^) and anorectic participants (with underweight BMI ≤ 17.5 kg/m^2^ and weight recovered BMI ≤ 18.5 kg/m^2^) and reasoned the following hypotheses. First, based on past studies we suggest elevated basal cortisol levels in P_AN_ in the resting condition (H_1_). This hypothesis relies on predominant literature reporting elevated basal levels (at rest) in P_AN_ (e.g., hypercortisolemia) even after treatment/weight recovery. Second, we expect significant group differences in HPAA reactivity. Thus, we hypothesized that P_AN_ would show a blunted hormonal pattern after stress exposure (TSST). Consequently, AUCi in HC will exhibit a significant increase compared with P_AN_ (H_2_). This hypothesis is based on past studies related to the HPAA functionality in P_AN_ suggesting abnormal functionality of the HPAA in P_AN_, as evinced by blunted reactivity to the TSST^28,32–34^ and also reflected in a diminished sensitivity to negative feedback to the DST^27,28^ and hypercortisolemia (e.g.,^10,11^). HPAA dysfunctions in P_AN_ has been often associated with chronic stress^8,17–19^, pronounced ED-symptoms^35,42^, and psychopathology (e.g.,^36–38^). Since P_AN_ in our study exhibit elevated basal cortisol levels, chronic stress, and psychological burden (compared with HC), a decreased sensitivity of the HPAA to negative feedback can be assumed. Hence it is conceivable that P_AN’s_ endocrine system may not perceived acute stress (VAS) (e.g., TSST) as stressful as compared with HC with lower basal cortisol. Therefore, we assume differences in HPAA reactivity between HC and PAN. Furthermore, most of the studies suggest that HPAA functionality is restored after weight recovery. Therefore, we reasoned that our results will support influential literature on the normalization of the HPAA activity in terms of total cortisol output and reactivity with weight recovery (H_3_; e.g.,^46,59^). Hence, we expect a positive correlation between BMI and AUC_i_ and a negative correlation between BMI and AUC_G_ in P_AN._

Laboratory studies measuring the neuroendocrine responses to a standardized stress task in ED patients and HC is relevant in determining whether AN is characterized by a general dysregulation of the autonomic stress response or not and might help in clarifying inconclusive results. To our knowledge this is the first study that analyses the endocrine response to acute psychosocial stress in a larger group of only P_AN_ under highly standardized conditions.

## Material and methods

### Study participants

In the present study, *n* = 26 participants with AN (*n* = 18 with a BMI > 18.5 Kg/m^2^ and *n* = 8 with a BMI < 17.5 Kg/m^2^) and *n* = 26 healthy participants (BMI = 23.08 ± 3.3 kg/m^2^) between 18 and 65 years of age were recruited through online media, newspapers, and bulletin boards at several universities. During the course of the study, P_ANs_ were still inpatients at the Polyclinic for Psychotherapy and Psychosomatic in Dresden, Germany, who were diagnosed with AN at the time of admission. During their inpatient treatment some of these patients were asked to participate in our study, but first after reaching a certain level of stability, which was reflected in their BMI (some of the patients were artificially nourished). Please see Table 1 for details. Still, P_AN_ with a higher BMI were also significantly taller (*M* = 170 ± 5) than P_AN_ with underweight (*M* = 162.4 ± 5.5). All P_AN_ were re-screened and included in the study after a positive examination of the inclusion criteria. The patients met all diagnostic criteria of AN according to the Structured Clinical Interview (SCID) for the Diagnostic and Statistical Manual of Mental Disorders (DSM-IV^74,75^). However, with the exception that some of the P_AN_ have recovered some weight during the course of inpatient treatment at the clinic and showed a BMI larger than 17.5 Kg/m^2^ (see Table 1). Exclusion criteria for the clinical group was the presence of other mental disorders (besides depression), health diseases, and intake of any type of medication. HC with a past or a current eating or other mental disorder, a chronic illness, medication treatment, stressful life events in the past 6 months and an abnormal BMI were excluded. HC were age and gender matched to P_AN_. All participants received an expense allowance of 50 Euro after successful participation on both test days. A description of all *N* = 52 study participants is provided in Table 1. All participants gave a written informed consent. Ethical approval was obtained from the Ethics Committee of the Medical faculty of the Technical University of Dresden, Germany (No#EK25012013).

**Table 1:** Characteristic of the matched participants.

	P_AN_				HC	*t*/*x*^2^	*p*	
Total *n* (%)	26					26		
Female	24 (92.3)					24 (92.3)		
Male	2 (7.7)					2 (7.7)		
Contraceptives	0 (100)					6 (25)	9.474	0.003^***.^
Cigarettes/tag	4.80 (7.07)					1.20 (3.25)	23.126	0.000^***.^
			*t*	*p*				
*M* (*SD*)	P_AN_ BMI							
>18.5	P_AN_ BMI							
<17.5								
BMI	20.7 (3)	16.0 (1)	4.34	0.000^***^		23.08 (3.3)	−4.139	0.000^***.^
Age	26.50 (6.11)	25.13 4.79	0.560	0.58		25.0 (5.5)	0.747	0.500
Size	170.00 (5.00)	162.38 (5.55)	3.30	0.003^*^		168.21(7.05)	0.452	0.653
*Psychological variables*			*t*	*p*				
TICS-9	2.00 (.85)	1.72 (.68)	0.826	0.417		1.12 (.51)	4.254	0.000^***^
					*Norm values*			
BDI	52.12 (29.70)	35.42 (23.29)	1.32	0.20	<14 = normal. 14–19 = mild. 20–28 = moderate. 29–63 = severe depression.			
SCL (GSI)	15.00 (9.00)	11.25 (5.36)	1.07	0.29	Score 11 = Percentile 92%. Score 15 = Percentile 95.2%			
EDI	154.166 (64.40)	156.00 (45.91)	0.072	0.93	Score 155 = Percentile = 85%.			
PASA (SI)-_R_	−1.04 (1.70)	−1.80 (1.58)	1.05	0.30				
PASA (SI)-_TSST_	0.74 (1.71)	0.71 (.97)	0.211	0.83				
VAS-_R_	37.00 (11.30)	39.20 (15.40)	−0.43	0.66				
VAS-_TSST_	55.25 (16.0)	56.40 (6.30)	−0.192	0.85				
AUC_G_-_R_	503.57 (30.30)	477.83 (184.84)	0.22	0.82				
AUC_G_-_TSST_	550.02 (273.48)	825.71 (558.559)	−1.71	1.00				
AUC_I_-_R_	−52.05 (246.44)	32.79 (139.249)	−0.90	0.37				
AUC_I_-_TSST_	111.65 (202.45)	250.56 (479.51)	−1.05	0.30				

*P*_AN_ participants with Anorexia nervosa, *HC* healthy controls, *M* mean, *SD* standard deviation, *TICS* trier inventory chronic stress, *BDI* beck depression inventory, *EDI* eating disorder inventory, *SCL* Symptom-Checklist-K-9, *PASA (SI)-R* Primary Appraisal Secondary Appraisal (Stress Index) in Resting Condition, *PASA (SI)-TSST* primary appraisal secondary appraisal (Stress Index) in stress condition, *VAS-R* visual analogue scale in resting condition, *VAS-TSST* visual analogue scale in stress condition.

***p ≤ 0.001; *p ≤ 0.05.

### Procedures

**Fig. 1:**
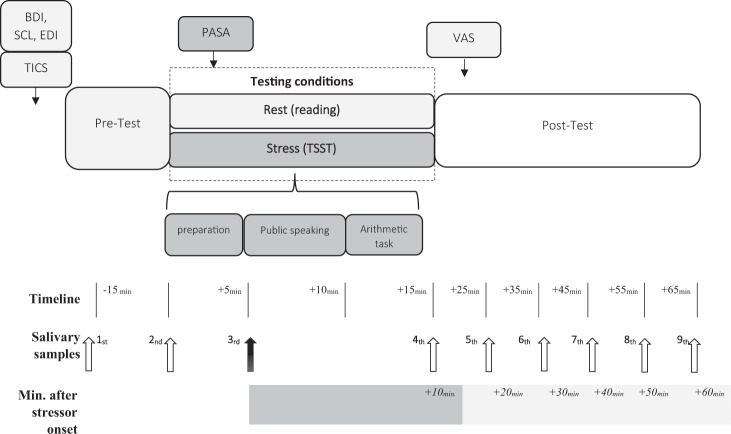
Timeline and design of experimental conditions. Trier Inventory for the assessment of Chronic Stress; PASA Primary Appraisal Secondary Appraisal questionnaire, VAS Visual Analogue Scale, BDI Beck Depression Inventory, SCL Symptom Check List-K-9, EDI Eating Disorder Inventory, TSST Trier Social Stress Test.

P_AN_ (*n* = 26) and HC (*n* = 26) were scheduled on consecutive days for two different experimental conditions (stress and rest) on a given weekday. The procedures took place between 2:00 and 4.00 p.m., in order to standardized cortisol measurements affecting its circadian rhythm. In addition, all healthy females and a small part of P_ANs_ (*n* = 5) were submitted to the TSST only during the luteal phase, since some of the patients were amenorrhoeic (*n* = 8) and some didn’t submit any information about their cycle (*n* = 13). Throughout the entire experiment a total of nine salivary samples were collected. Upon arrival, participants signed an informed consent form and completed psychological measures. The acclimatization phase of the participants lasted around 15 min. Acclimatization of the participants to the laboratory settings facilitate pre-study cortisol levels to return to baseline^76^, thereby allowing a strong TSST responses and low inter-individual variability^77^. The testing sequence was randomized: 26 participants initially completed the resting condition, while the other 26 started with the stress condition. On the next day, all participants returned to complete the remaining condition. The *stress* condition consisted on the exposure to an acute stressor (TSST), while during the *resting* condition participants read in quiet and in a neutral environment. The entire experimental procedure took around 90 min and was divided in three phases: (1) pre-test, (2) intervention phase (*rest* or *stress*) and (3) post-test. The experimental conditions were set as follows. (1) During the pre-test phase (~30 min.), all participants completed psychological questionnaires. Salivary samples were collected 15 min. prior intervention (*stress* or *rest*) and immediately at the start. prior intervention. (2) In the following 15 min, participants were *either* exposed to the *stress* (TSST) or *resting* condition (quiet reading/room). At the beginning of the assigned condition, all participants filled out the Primary Appraisal Secondary Appraisal (PASA) for the purpose of evaluating stress perception elicited by the corresponding condition. Participants, who were submitted to the *stress* condition (TSST) underwent three test phases (5 min each = 1. preparation, 2. interview, 3. math task). The *resting* condition took place in a secluded and quiet laboratory room, where fordable literature was suggested to counteract possible superfluous arousal effects. At this stage, three salivary samples were collected every 10 min. (3) Immediately after the assigned condition (either *stress* or *rest*), the participants completed the Visual Analog Scale (VAS) for subjective stress perception assessment with reference to the respective condition. Along the post-test, five salivary samples were collected every 10 min. Please see Fig. 1 for an overview of the procedure.

### Cortisol samples and analyses

Cortisol concentrations were measured by means of salivary samples collected by a Salivette^®^ (Sarstedt, Nümbrecht, Germany). This procedure consists in salivary moistening of a cotton roll for 1 min and placing it into a salivette-device immediately afterwards. Salivettes were refrigerated at 2–8 °C. Salivary cortisol analyses were run after centrifugation using the luminescence immunoassay test method. The latter procedure provides an intra- and inter-assay coefficient of variation below 9.0%, which is considered as robust and valid^78^.

### Stress intervention

*Trier Social Stress Test* (TSST^79^). The TSST is known for its highly standardized protocol procedure and its effectiveness in eliciting a stress reaction. This protocol was applied in the stress condition for laboratory stress induction purposes. The TSST is also an internationally established test for inducing an acute psychosocial stress response and its effectiveness has been demonstrated in countless studies worldwide^76,80^. It conveys uncontrollability, social evaluation, and arithmetic tasks. Subjective perception and cognitive evaluation of stress was assessed on account of the scales PASA and VAS, which were submitted during and after the 15 min stress test.

### Psychological assessments

*Trier Inventory for Chronic Stress* (TICS-9^81,82^). This scale is based on a larger version conveying 57 items and was applied to measure perceived chronic stress during the last 3 months. The short version consists of nine items representing the main dimensions of the long version (i.e., work and social overload, pressure to perform, work discontent, excessive demands at work, lack of social recognition, social tensions, isolation and chronic worrying). Item ratings range from 0 to 5 (0 = never, 1 = rarely, 2 = sometimes, 3 = often, 4 = very often). Higher values indicate greater stress. Satisfactory psychometrics have been demonstrated in many studies and exhibits good reliability between Cronbach’s Alpha *α* = 0.88–0.91^82–84^.

*Primary Appraisal Secondary Appraisal* (PASA^85^). This measure evaluates four cognitive appraisal processes *threat*, *challenge*, *self-concept of own abilities*, and *control expectancy* based on a six-point rating scale. Relying on the four primary scales two secondary scales and a stress index are calculated. *Primary appraisal* encompasses *threat* and *challenge*, while *secondary appraisal* includes *self-concept of own abilities* and *control expectancy*. The *stress index* is calculated by subtracting the primary appraisal from the secondary appraisal and offers a global assessment. This scale was applied to evaluate stress perception (acute stress intervention). The scale has shown satisfactory psychometric values^86^.

*Visual Analog Scale* (VAS^87^). This tool serves as a visual aid scale and it is widely applied to quantify self-report instruments. In our study it was employed immediately after both experimental conditions (stress vs. rest) to rate stress perception from 0 (no stress) to 100 (maximum stress). Satisfactory psychometric properties and usefulness and economy have been evinced in a variety of studies^87–89^.

*Symptom-Check-List-9* (SCL-K-9^90^). This scale is a short version of the Symptom-Checklist-90-Revided^91^, which is one of the most applied self-rating scales for assessing general psychopathology. It comprises 90 items to be rated on a 5-point Likert scale (from 0 = never to 4 = very often), which indicate symptom severity in nine main psychopathological dimensions: *somatic symptoms, interpersonal sensitivity, obsessive–compulsive behaviors, anxiety and depressive symptoms, hostility, phobic symptoms, paranoid tendencies*, and *psychoticism*, and are also comprised in the short version. The scale also provides a global severity index (GSI) as an indicator of overall psychological distress, with higher scores reflecting higher levels of psychopathological distress as well as a greater symptom severity. For the purpose of the present study, we only reported GSI-values. The sum score is calculated by addition of the scores of all scales. The psychometric properties are satisfactory and its reliability values range from α = 0.83 to 0.8792^92–94^. Normative percentile values specific to age and gender are provided^94^.

*Beck Depression Inventory* (BDI^95,96^). This scale was applied to measure depressive symptoms in P_AN_. The BDI is a self-report questionnaire consisting in 21 items, which describe symptoms and attitudes to be rated in terms of intensity from 0 to 3 (total score range: 0–63). The total score is calculated by addition of the items. Higher scores indicate greater severity of symptoms. Cut-off values are established as follow: <14 = normal, 14–19 = mild depression, 20–28 = moderate depression, 29–63 = severe depression. The psychometric properties of the BDI are satisfactory. Cronbach’s Alpha ranges from *α* = 0.89 to 0.94^97,98^.

*Eating Disorder Inventory* (EDI^99,100^). The EDI was applied to measure ED related symptoms and attitudes relevant to pathological eating behavior. The scale is composed of 64 items comprised in 8 dimensions: *Drive for Thinness*, *Bulimia* (B), *Body Dissatisfaction* (BD), *Ineffectiveness* (I), *Perfectionism* (P), *Interpersonal Distrust* (ID)I *Interoceptive Awareness* (IA), and *Maturity Fears* (MF). The items are to be rated on a six-point rating scale (0 = never to 5 = always). Reported Cronbach’s Alpha range from α = 0.72 to 0.92^100,101^. The values of the P_AN_ in the present study are based on the percentile values provided^102^.

### Statistical analysis

The optimum statistical sample size was calculated with the G*power program (version: 3.1.9.2.). Based on a medium effect size of Cohen’s *f* = 0.25, two groups (HC vs. P_AN_), *n* = 8 repetitions, a significant level of *p* = 0.05, power of 80% (1–*β* = 0.80), and after Bonferroni-correction, a total sample size of *n* = 26 for within-subjects factor and *n* = 52 for between-subjects factor was needed. For the analyses of the cortisol output of anorectic adults and healthy controls after a stress condition. A 2 _(condition: rest vs. TSST)_ × 8 _(time)_ × 2 _(PAN vs. HC)_-factorial ANOVA for repeated measures with *time* and *condition* as within-factor and *group* as between-factor was calculated with SPSS (version 26). The assumption of sphericity was controlled by Mauchly’s test, and if required, the Greenhouse-Geisser correction was applied. Prior to data analyses, participants were first matched for age and gender. In the next step, sociodemographic and life-style variables (e.g., smoking and use of contraceptives) between both groups were examined by means of the independent *t*-test and chi-square test. Second, the cortisol pathway response after stress induction in both groups (over eight measurement points: −5, +0, +10, +20, +30, +40, +50, +60) were analyzed by a two-factorial ANOVA for repeated measures with the between-factor *group* (*N* = 52) and the within-factor *time* (*n* = 26). The pre-test measure of −15 min was not included in the analyses, since elevated pre-test cortisol levels could possibly mask laboratory induced stress, resulting in falsely altered responses^77^. Use of oral contraceptive and smoking status were tested to control influencing factors on cortisol. Since cortisol data was not normally distributed, we worked with values subjected to log transformations. Moreover, the following cortisol parameters were calculated: area under the curve (AUC) with respect to ground (AUC_G_) and increase (AUC_I_), as well as reactivity and peak-base values were calculated. Reactivity is defined as the change in salivary cortisol during the experimental phase and is calculated as the difference between the first and the last sample, while peak-base is the time from baseline to peak-value^103^. An ANOVA was further computed to evaluate differences in the derived cortisol parameters, in basal levels and stress appraisal between the research groups. To specify the cortisol response to the TSST, the participants were categorized into non-responders and responders based on an increase of at least 1.5 nmol/L^104^.

Subsequently, a correlation was performed between BMI and AUC_G_, and BMI and AUC_I_ values in order to investigate the relationship between the cortisol and BMI in P_AN_. Thereafter, a further *t*-test for independent samples was computed to assess group differences (P_AN_ vs. HC) in chronic stress (TICS-9) and further stress appraisal scales (PASA and VAS). Moreover, psychological measures (EDI, BDI, SCL-K-9) in P_AN_ were evaluated and compared with available norm values. For exploratory purposes we computed correlations (Pearson-Product-Moment-Correlations) between BMI values and psychological measures. Lastly, we calculated *t*-test to assess the data of only P_AN._ We compared the data of underweight anorectic patients (BMI < 17.5 Kg/m^2^) to weight recovered patients (BMI > 18.5 Kg/m^2^).

## Results

### Psychological measures

A summary of the sociodemographic variables of all study participants is displayed in Table 1. In terms of randomization of the procedure, there was no sequence effect. P_AN_ and HC were successfully matched for age and gender. Significant differences between the two groups in terms of BMI use of contraceptives, smoking, and chronic stress were exhibited. As observed, none of the members of the clinical group used contraceptives, but smoked significantly more than the healthy controls. In addition, P_AN_ reported higher values of chronic and perceived stress, as well as depression in comparison to HC and norm values respectively. In addition, P_AN_ showed pronounced values in terms of general psychological distress (GSI) and ED symptomatology (EDI) according to percentile values (see Table 1). Since some of the P_AN_ had recovered some weight (BMI > 17.5 Kg/m^2^), while others were still underweight (BMI < 17.5 Kg/m^2^) we re-analyzed data comparing the above-mentioned group categories in P_AN._ Further analyses (e.g., *t*-tests) showed neither significant differences between the two groups in terms of psychological measures nor in cortisol (see Table 1). As shown in Table 2, VAS after stress induction was reflected in values higher than 0 without a significant difference between both groups (*t*_(1, 50)_ = 0.195; *p* = 0.71), indicating a successful stress induction. In terms of appraisal (PASA), significant differences between the groups were exhibited only in particular subscales: *Threat* (*t*_(1, 50)_ = 2.101, *p* = 0.041), *Self-Concept t*_(1,50)_ = −4.802, *p* = 0.000), and *Secondary Appraisal* (*t*_(1, 50)_ = −3.332; *p* = 0.002) as well as in the *Stress-index* (*t*_(1, 50)_ = 2.750; *p* = 0.008), whereby P_AN_ showed a higher stress index. PASA-values were correlated with different cortisol parameters, however no significant correlations were shown (e.g., AUC_G_ and PASA (SI), *r* (25) = −0.108, *p* = 0.608; AUC_G_ and PASA (SI), *r* (25) = −0.199, *p* = 0.339). In general, P_AN_ evaluated the experimental procedures as more threatening and experience themselves as less influential over the ongoing circumstances. Further, they felt less capable of coping with the given situation than healthy adults (see Table 3). Additional analyses revealed significant correlations between BMI and BDI (*r* (25) = 0.402, *p* = 0.046), but not between BMI and EDI (*r* (25) = 0.332, *p* = 0.098) or BMI and GSI (*r* (25) = 0.323, *p* = 0.107). This means that BMI is rather associated with depression rather than with ED-symptoms or distress. Significant and high correlations were also observed between EDI and GSI, *r* (26) = 0.733, *p* = 0.000, EDI and BDI *r* (25) = 0.664, *p* = 0.000, but not between EDI and BMI *r* (26) = 0.332, *p* = 0.098. These results suggest that EDI is rather associated with distress and depression rather than with BMI. Reactivity correlated high with EDI *r* (26) = −0.505, *p* = 0.008 and moderately with GSI *r* (26) = −0.392, *p* = 0.048, but not with BMI *r* (26) = −0.114, *p* = 0.56. Therefore, the higher the ED-symptoms and distress, the weaker the reactivity. In conclusion these outcomes propose that reactivity is rather related to ED-symptoms and psychological burden than to BMI.

**Table 2:** Cortisol parameters and Subjective Appraisal in P_AN_ and HC - Conditions and Groups.

	P_AN_		HC		ANOVA								
*M* (*SD*)	Condition		Condition		Condition			Group			Interaction		
AUC	Rest	Stress	Rest	Stress	*F (df)*	*p*	*η*^*2*^	*F*	*p*	*η²*	*F(df)*	*p*	*η*^*2*^
AUC_G_	495.65 (266.49)	634.85 (393.76)	372.41 (171.98)	693.12 (330.65)	39.19 (1, 50)	.000^***^	.439	.086(1, 50)	.770	.002	7.19 (1, 50)	0.010^*^	0.126
AUC_I_	−25.94 (219.83)	154.393 (310.68)	8.94 (114.98)	312.37 (342.07)	20.91 (1, 50)	.000^***^	.295	20.66(1, 50)	.015^*^	.113	2.96 (1,50)	0.091	0.056
**Baseline** (t-15 min.)	11.53 (6.80)	11.00 (6.00)	7.49 (3.11)	8.68 (4.44)	177 (1, 50)	.676	.004	6.54 (1, 50)	.014^*^	.116	1.22 (1, 50)	0.274	0.024
(t-1 min.)	10.22 (6.41)	9.42 (5.21)	7.12 (3.71)	7.46 (4.00)	.076 (1, 50)	.784	.002	5.48 (1, 50)	.023^*^	.099	.454 (1, 50)	0.503	0.009
**Subjective Appraisal**													
PASA - SI	−1.38 (1.62)	.70 (1.49)	−2.40 (.98)	−.23 (.87)	120.508 (1, 49)	.000^***^	.711	10.59 (1, 49)	.002^**^	.178	.045 (1, 49)	0.833	0.001
VAS	37.39 (12.62)	55.62 (13.49)	30.49 (10.72)	54.89 (13.22)	114.205 (1, 49)	.000^***^	.700	1.737 (1 49)	.197	.034	2.39 (1, 49)	0.128	0.047

*P*_AN_ all participants with Anorexia nervosa, *HC* healthy controls, *M* Mean, *SD* standard deviation, *AUCG* area under the curve with respect to the ground, *AUCI* area under the curve in respect to the increase, *PASA* primary appraisal secondary appraisal, *VAS* visual analogue scale.

*p ≤ 0.05; **p ≤ 0.01; ***p ≤ 0.001.

**Table 3:** Influence of the stress condition on subjective appraisal and hormonal response in P_AN_ and HC.

	P_AN_	HC	*t*	*p*
PASA	*M* (SD)			
*Subjective appraisal*				
Threat	4.30 (1.15)	3.72 (0.788)	2.10	0.041*
Challenge	4.59 (0.97)	4.58 (0.533)	0.01	0.987
Self-concept	2.93 (1.11)	4.20 (0.74)	−4.80	0.000^***^
Control expectancy	4.55 (0.68)	4.57 (0.868)	−0.10	0.915
Primary appraisal	4.44 (0.98)	4.15 (0.57)	1.28	0.206
Secondary appraisal	3.74 (0.70)	4.40 (0.68)	−3.33	0.002^***^
VAS	55.62 (13.49)	54.89 (12.22)	0.19	0.84
*Hormonal response*	*M (SD)*		*t*	*p*
*Derived parameters*				
Peak-base	7.5 (8.53)	10.7 (9.04)	−2.25	0.029*
Reactivity	0.73 (5.10)	4.25 (6.30)	−2.17	0.036*

*P*_AN_ all participants with Anorexia nervosa, *HC* healthy controls, *M* mean, *SD* standard deviation, *PASA* primary appraisal secondary appraisal, *VAS* visual analogue scale.

*p ≤ 0.05; **p ≤ 0.01; ***p ≤ 0.001.

### Cortisol response and total output

Due to a skewed distribution of the cortisol data, statistical analyses were computed on log-transformed values. The pre-test measure of −15 min was not included in the analyses, since pre-test cortisol levels were elevated in spite of the acclimatization phase (as recommended by 78 and 79) and including it may result in falsely altered responses^77^. Cortisol values and further derived parameters are displayed in Tables 2–4. There were no significant differences between responders and non-responders (*z* = −0.363, *p* = 0.717).

**Table 4:** Cortisol values of P_AN_ and HC in nmol/l at nine measurement timepoints.

Salivary samples	^a^Min.	Rest	Stress	Condition	Rest	Stress
		P_AN_			HC	
		*M* (SD)			*M* (SD)	
1	−20	11.53 (6.80)	11.00 (6.00)		7.49 (3.11)	8.68 (4.44)
2	−5	10.22 (6.41)	9.42 (5.21)		7.12 (3.71)	7.47 (4.00)
3	0	10.23 (6.33)	9.51 (4.77)		7.46 (3.77)	8.25 (3.88)
4	10	9.91 (6.28)	11.84 (8.37)		7.34 (3.49)	10.98 (5.74)
5	20	8.88 (5.42)	13.88 (9.99)		6.66 (3.45)	16.51 (9.02)
6	30	9.73 (6.18)	14.24 (10.52)		7.32 (4.00)	14.31 (8.24)
7	40	10.28 (6.07)	12.28 (8.68)		7.42 (3.63)	13.77 (6.43)
8	50	10.03 (5.18)	11.36 (5.56)		7.68 (3.73)	13.01 (6.29)
9	60	9.17 (5.17)	10.15 (5.63)		7.38 (3.69)	11.71 (5.73)

*P*_AN_ all participants with Anorexia nervosa, *HC* healthy controls, *M* mean, *SD* standard deviation.

^a^Min. relative to stressor onset.

As predicted (H_1_), baseline cortisol levels at rest (*resting condition*) were higher in P_AN_ in contrast to those of healthy individuals, while no sig. baseline differences were found in the stress condition (see Table 2).

In terms of the cortisol response, a 2 (_condition: rest vs. TSST_) × 8 (_time: −5, +0, +10, +20, +30, +40, +50, +60_) × 2 factorial ANOVA for repeated measures with *time* and *condition* as within-factor and *group* effect (P_AN_ vs. HC) as between-factor revealed the following results. A *group* effect was not observed *F* (1, 50) = 0.69; *p* = 0.410; $\eta _p^2 = 0.014$). This means that both groups exhibited comparable cortisol mean values. Further analyses showed a highly significant *condition* effect (*F* (1, 50) = 20.50; *p* = 0.000; $\eta _p^2 = .291$) accounting for almost 30% of the variance in participants’ cortisol values. Moreover, a highly significant effect of *time* over the eight measurement points was evident (*F* (2.71, 135.44) = 11.27; *p* = 0.000; $\eta _p^2 = 0.20$). Further, two significant interaction effects were observed (1. *Condition* × *group* and 2. *Condition* × *time*). First (marginally significant), *condition* × *group*: (*F* (1, 50) = 4.17, *p* = 0.046; $\eta _p^2 = 0.077$), indicating that regardless of *time*, significant variation in cortisol was observed as a function of both, condition and group. Second (highly significant), *condition* × *time: F* (2.71, 135.44) = 16.07, *p* = 0.000, $\eta _p^2 = 0.24$. This outcome indicates significantly different cortisol response patterns. All participants demonstrated higher levels of salivary cortisol after the stress intervention, than during the resting condition. When controlling for oral contraceptive use, the effect of *condition* and *group* on cortisol was attenuated but remained significant. In spite of that, the effect of factor *time* on cortisol was marginally affected by cigarette smoking (*p* = 0.045; $\eta _p^2 = 0.064$), but not by oral contraceptive use. As aforementioned, P_AN_ smoked significantly more than HC.

**Fig. 2:**
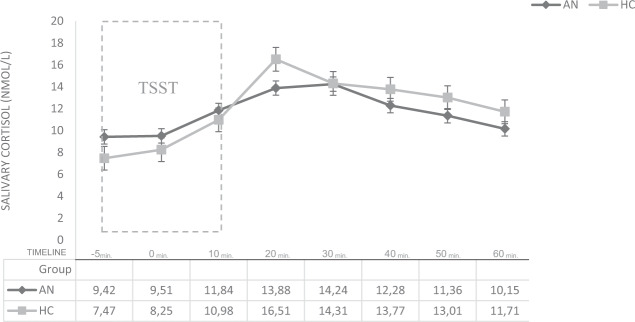
Cortisol increase in terms of AUCi. Significant cortisol increase in PAN vs. HC in the stress condition *p* > 0.001. PAN individuals with Anorexia Nervosa, HC healthy controls.

In terms of reactivity, both groups (HC and P_AN_) showed a TSST-induced cortisol response. However, as predicted (H_2_), HC demonstrated a higher increase in salivary cortisol (to 221.313% of the baseline) than P_AN_ (151.167%)—see Fig. 2. Also, in terms of an increase (AUC_I_), significant differences were revealed between both groups (HC vs. P_AN_)—see Table 2. This increase was also clearly visible. As illustrated in Fig. 2, healthy participants reacted with a greater cortisol increase (from baseline to peak, +20 min. post-stressor onset: *t*_5_ = 16.51 (9.02), *p* = 0.029) and reactivity (*M*_PAN_ = 0.73 vs. *M*_HC_ = 4.25, *p* = 0.036) to the stress intervention, when compared with the patient group. As assumed (H_2_), these results indicate altogether a blunted cortisol reactivity in P_AN._ Further, our data notably shows (see Table 4) a delayed cortisol response in P_AN_ (after 30 min.) from stressor onset (i.e., public speaking), when compared with the control group (after 20 min.)—as illustrated in Fig. 2. Concerning our last hypothesis related to P_AN_ (H_3_), we expected a significant inverse correlation between total cortisol output (AUC_G_) in the stress condition and BMI. The results confirm this hypothesis = *r* (24) = −0.42, *p* = 0.027. This outcome indicates that P_AN_ with a higher BMI released less total cortisol in response to the stress condition (AUC_G_). We also hypothesized a positive correlation between a cortisol increase (AUC_i_) and BMI. However, this could not be demonstrated = (*r* (24) = −0.26, *p* = 0.20). Please insert Fig. 2.

## Discussion

The present study investigated the salivary cortisol response to a psychosocial stressor under highly standardized laboratory conditions in inpatients with AN and a matched sample of healthy participants. Our data could replicate the results of many studies showing elevated basal levels in P_AN_ (H_1_) relative to HC. In terms of reactivity, both groups displayed an increase in cortisol after stress induction. However, HC showed a common cortisol response often seen in healthy adults (i.e., relatively rapid cortisol onset followed by decreased values with time progression), while P_AN_ exhibited a delayed and blunted cortisol response pattern (H_2_). Overall, no significant *group* (HC vs. P_AN_) differences were shown in total cortisol output. Furthermore, our data indeed suggests reversibility of hypercortisolemia with a higher BMI, but with persistent irregularities in cortisol reactivity (H_3_). Altogether, underweight anorectic (BMI < 17.5 Kg/m^2^) and weight recovered patients (BMI > 18.5 Kg/m^2^) show comparable results in terms of cortisol response, reactivity, and psychological measures. Concerning psychological variables, P_AN_ generally reported greater chronic and perceived stress relative to HC. P_AN_ also felt more threatened and less resourceful when confronted with the stress intervention, in contrast to HC. What is more, they reported pronounced symptoms of depression (BDI), psychological distress (GSI), and ED-symptomatology according to norm values. Additional analyses revealed significant correlations between BMI and depression, but not between BMI and EDI or GSI. Also, EDI and GSI as well as EDI and BDI correlated high. Reactivity correlated high with EDI and moderately with GSI but not with BMI. In conclusion these outcomes propose that ED-symptoms and reactivity are related to psychological burden than to BMI.

In summary, the outcomes in the present study reflect previous findings in the field of research concerning P_AN_ and reveal comparable outcomes related to the cortisol response in the context of studies using the TSST. In line with the majority of studies on cortisol in P_AN_ (as previously described), our data could also demonstrate elevated basal cortisol levels in AN at rest. Therefore, our first hypothesis could be confirmed. Further, our study was able to replicate the results of Het et al.^32^ and Zonnevylle-Bender et al.^34^ showing a blunted HPAA reactivity to acute stress (TSST). The latter was also evinced by Vocks et al.^33^, however stress exposure was implemented by responding to a mirror task. Notwithstanding, Monteleone et al.^68^ reported a preserved cortisol response to the TSST in P_AN_, however this occurred at significantly higher hormone levels (pre-and-post stressor). Possible sources of discrepancies between this outcome and our findings might arise as a result of methodological dissimilarities, including sample size, patient populations, and cortisol assessments (e.g., confounding variables), as revised in the meta-analyses of Monteleone et al.^73^. Notable difference in sample sizes and mixed clinical groups, are for example noticed in Monteleone’s^68^ study. In such, only seven patients with were compared with eight HC, making results susceptible to random fluctuations. A further explanation may lie in the characteristics of the P_AN_. As mentioned, our participants were inpatients still receiving intensive treatment and the majority (*n* = 18) had a larger BMI (≥17.5–19 kg/m^2^) than normally observed in affected subjects (BMI < 17.5 Kg/m^2^). On the other hand, the participants in the study of Monteleone et al.^68^ were outpatients with a quite low BMI (*M* = 16.3, *SD* = 1.2) and possibly malnourished (starvation-mode). The effects of starvation are well known for triggering HPA-axis hyperactivity (e.g.,^25,105^), resulting in higher cortisol levels. Such were indeed evident in P_AN_ throughout their entire experiment, when compared with HC. The researchers assumed a hyperactivity of the HPAA, but with a “preserved” reactivity^68^. Considered by itself, this pattern could be interpreted more as an irregularity, than an average response to stress. A common response to stress as presented in healthy individuals, is characterized by a rapid onset followed by a quick recovery^76,79^. This pattern common to HC was also displayed by the control group described in the current study and is line with responses reported in a recent metanalysis^77^. What is more, cortisol assessment could have been probably affected by the participants’ menstrual cycle. The female participants in our study were tested in their luteal phase, however many of the P_ANs_ did not provide data on their cycle and some were amenorrhoeic, while participants in Monteleone’s^68^ study were tested during their follicular phase. It is assumed that stress responsivity varies across the menstrual cycle^75–77^. Beyond that, P_AN_ in our study smoked significantly more than HC, which have may affected cortisol levels, since smoking affects the endocrine system^106^.

After all, further considerations may be plausible in terms of the blunted cortisol response pattern seen in P_AN_ in our study. As reported by past studies, besides caloric restriction/starvation, other variables affect HPAA functionality e.g., chronic stress, of childhood adversities, psychological burden, and pronounced eating pathology. As aforementioned, the P_AN_ in the present study were still in therapy and demonstrated relatively high levels of chronic and perceived stress, as well as psychological burden and ED-symptoms. Thus, it is conceivable that this increased level of chronic stress together with the pronounced threat perception (PASA) and psychopathology (BDI, GSI) accounted for HPAA dysregulation in P_AN_ to a certain extent. It is known that stress appraisal is a relevant determinant of the cortisol response^85^ and chronic stress results in long-lasting HPAA activity dysregulation^107^, e.g., attenuated reactivity resulting in a blunted hormonal response^51,65^. Correspondingly, Lelli et al.^35^ reported that patients with a history of childhood adversities (CHA) exhibited an attenuated cortisol response before and after therapy. Analogous results were presented in the research of Monteleone et al.^71^: P_AN_ with CHA demonstrated a blunted cortisol response to the TSST as compared with controls (HC and P_AN_ without CHA), but with a comparable amount of overall cortisol production, as in the case of the present experiment. Hence, we speculate that the P_AN_ in our study, have also had been affected by adverse experiences in the past. This assumption is based on the blunted response, pronounced ED-symptoms and psychological burden (e.g., GSI, BDI) even while being treated. However, psychological burden alone is also related with a blunted cortisol response to acute stress^36,38–41^ as presented in P_ANs_ in our current study. Therefore, it is conceivable that the observed symptomology (i.e., pronounced chronic and perceived stress, ED-symptoms, depression and psychological distress) might have accounted for the blunted response to the TSST.

Furthermore, this blunted cortisol response in P_AN_ was evident in spite of a higher BMI, since P_AN_ are mostly underweight (BMI < 17.5 Kg/m^2^). In relation to this parameter, we predicted that irregularities in hormonal response to an acute stressor (TSST) are likely to normalize in individuals with a higher weight. Therefore, we expected an inverse correlation between BMI and AUC_G_ and a positive correlation between BMI and AUC_I_ and (H_3_). A significant inverse linear correlation between total cortisol output (AUC_G_) and BMI was indeed observable, suggesting reversibility of hypercortisolemia with weight recovery. Still, HPAA irregularities in terms of reactivity persisted. Thus, this hypothesis was only partially confirmed. In the present study, a lack of hypersecretion of total salivary cortisol output was also manifested in similar AUC_G_-values between P_AN_ and controls, since total cortisol output was comparable (also confirmed by the 2 × 2 × 8 ANOVA). Similarities in total cortisol output were also reported in comparable studies^32,34,71^. The former finding is also in accordance with abundant studies reporting a normalization of hypercortisolemia (e.g.,^60,64,108,109^) in weight recovered P_ANs_. Likewise, earlier studies support our outcome^58^, e.g., Walsh et al.^29^ examined the adrenocortical activity in underweight and weight recovered anorexia patients during a 24 h period. The results revealed that as anorectic patients recovered weight, the rate of cortisol production decreased. Other researchers have also replicated this outcome. These finding proposes that dysregulated activity of the HPAA of symptomatic P_AN_ is related to extreme emaciation or underweight. Consequently, it is concluded that altered activity of the HPAA in P_AN_ in terms of total cortisol release is a state-dependent phenomenon. On the other hand, our results did not confirm that reactivity normalizes with weight gain (non-sig. AUC_I_). The results therefore suggest that the HPAA response to acute stress is still affected in P_AN_. Therefore, we assume that hypersecretion of cortisol does attenuate with a higher BMI, but irregularities in terms of reactivity persist (even at a BMI = 19Kg/m^2^). Nevertheless, P_AN_ had still notable ED-symptoms and were still receiving inpatient treatment. In addition, full realimentation (in the sense of weight restorage) and recovery was not concluded. Consequently, it is not clear whether this pattern may change after full remission. In conclusion, this outcome implies that HPAA functionality is not fully restored after weight recovery and that functionality is sensitive to different types of stressors: e.g., bodily stressors (e.g., starvation) acutely affect cortisol production, while chronic stressors affect reactivity long-term. This suggest, that even though hormonal alterations are reversed by refeeding, weight recovery does not restore the full range of HPAA functionality^110^. Karin et al. recently proposed a detailed explanation of this phenomenon. The researchers elucidated that prolonged HPA activation enlarges the functional masses of the pituitary corticotropes as well as adrenal cortex and that their recovery from stress takes weeks even after stress cessation. In short, this mechanism clarifies why ACTH responses remain blunted for weeks even after cortisol production is normalized/regulated. This explains the normal cortisol output (AUC_G_) with a higher BMI, despite blunted reactivity in our sample of P_AN_.

Overall, these findings raise the question whether other variables besides BMI and chronic stress promoted maintenance of the blunted cortisol response in anorexia patients. As previously stated, massage therapy resulted in comparable hormone levels between P_AN_ and controls, without significant weight changes. Qualitative studies also claim that interventions aiming at weight changes are not beneficial for recovery in patients with an ED^111^. In this light, it seems reasonable to consider other psychological variables as influential factors, such as depression, distress, stress appraisal as well as coping skills. Indeed, our data showed that ED-symptoms are highly correlated with psychological distress and depression, but not with BMI. In addition, it could be demonstrated that reactivity is rather related to ED-symptoms and psychological burden than to BMI. As a matter of fact, recovered patients (weight remission), still experience poor well-being^112,113^ and low quality of life^113,114^ despite significant treatment response^112^ even after 2 years of therapy, compared with controls^115,116^. Particularly, fully recovered ED patients highlight factors such as social support and the development of new coping skills as essential to the process of (mental and physical) recovery^117–119^.

In summary, the main results of our study replicated past research showing elevated basal cortisol levels (at rest) and a blunted cortisol response in P_AN_ in response to acute stress exposure. In addition, our data emphasized that with weight gain, hypercortisolemia might be attenuated, but HPA-axis irregularities in terms of cortisol reactivity may persist. Additional analyses of our data attests past outcomes on the detrimental effects of chronic and perceived stress, as well as psychological burden on the HPAA activity. As such, this outcome is a major strength of the present study since it fosters understanding in the psychology of affected individuals and provides input for further potential designs aiming a differentiated analysis on the particular functionality of the HPAA in terms of cortisol production and reactivity. Importantly, it suggests that psychological burden has a higher impact on ED maintenance than weight itself. A further strength of our research were the highly standardized procedures and laboratory settings: Both investigated groups were clearly separated in normal-weight controls and anorectic individuals, who were perfectly age and gender match, excluding confounding factors by means of strict inclusion criteria. In addition, we employed a highly standardized psychosocial stress test (TSST) and methodological procedures for accurate cortisol assessment purposes, by taking confounders into account (e.g., menstrual cycle, smoking, contraceptive) and considering strict exclusion criteria (e.g., acute or chronic illness, mental disorders, medication, or substance intake). Notwithstanding, limiting factors are the lack of male participants (limited generalizability) and the lack of data related to measurement of illness duration, number of days at the clinic and childhood adversities. Future research might benefit from including this additional data in future studies. Thereupon, it remains to clarify whether HPAA reactivity can be completely restored, not only after weight recovery, but specially after full remission of psychological burden (e.g., depression and distress) and perhaps after regaining healthy levels of well-being.

The aim of the present study was to study the cortisol response of P_AN_ elicited by an acute stressor. A further purpose was to investigate the relationship between BMI and the cortisol response. In conclusion, compared with healthy controls, inpatients with AN evinced a blunted hormonal response to stress, which indicated alterations in the HPAA. On the other hand, our data supported partial reversibility of HPAA dysfunction: while cortisol output was regulated with weight recovery, irregularities in HPAA reactivity persisted. Based on our data we suppose that in addition to weight recovery, other psychological variables might affect HPAA reactivity (e.g., stress appraisal, depression, and psychological distress). In addition, our study makes clear that weight recovery is not crucial for ED recovery. Even after weight recovery P_AN_ still deal with ED-symptoms, depression, and psychological distress. Consequently, it is advisable to address psychological burden in therapy sessions.
