# Correction: Cortisol reactivity in patients with anorexia nervosa after stress induction

**DOI:** 10.1038/s41398-021-01326-6

**Published:** 2021-04-08

**Authors:** Ileana Schmalbach, Benedict Herhaus, Sebastian Pässler, Sarah Runst, Hendrik Berth, Silvia Wolff-Stephan, Katja Petrowski

**Affiliations:** 1 Medical Psychology and Medical Sociology, University Medical Center of the Johannes Gutenberg-University, Mainz, Germany; 2 Technische Universität Dresden, Carl Gustav Carus Faculty of Medicine, Division of Psychological and Social Medicine and Developmental Neurosciences, Research Group Applied Medical Psychology and Medical Sociology, Dresden, Germany; 3 University Hospital Carl Gustav Carus Dresden, Department of Psychotherapy and Psychosomatic Medicine, Dresden, Germany

Correction to: *Translational Psychiatry* (2020) **10**:275

10.1038/s41398-020-00955-7 published online 10 August 2020

Due to an error in typesetting, the values in Table 2 were not placed in the correct columns. This has now been corrected in the online and PDF versions of the article. Some minor corrections to references have also been made. The authors apologize for the mistakes. The original article has been corrected.

